# LogicNet: probabilistic continuous logics in reconstructing gene regulatory networks

**DOI:** 10.1186/s12859-020-03651-x

**Published:** 2020-07-20

**Authors:** Seyed Amir Malekpour, Amir Reza Alizad-Rahvar, Mehdi Sadeghi

**Affiliations:** 1 School of Biological Sciences, Institute for Research in Fundamental Sciences (IPM), Tehran, Iran; 2 National Institute of Genetic Engineering and Biotechnology, Tehran, Iran

**Keywords:** Gene regulatory network, Probabilistic logic, Fuzzy logic, Gene expression data, Bayesian information criterion (BIC), Bayes factor (BF)

## Abstract

**Background:**

Gene Regulatory Networks (GRNs) have been previously studied by using Boolean/multi-state logics. While the gene expression values are usually scaled into the range [0, 1], these GRN inference methods apply a threshold to discretize the data, resulting in missing information. Most of studies apply fuzzy logics to infer the logical gene-gene interactions from continuous data. However, all these approaches require an a priori known network structure.

**Results:**

Here, by introducing a new probabilistic logic for continuous data, we propose a novel logic-based approach (called the LogicNet) for the simultaneous reconstruction of the GRN structure and identification of the logics among the regulatory genes, from the continuous gene expression data. In contrast to the previous approaches, the LogicNet does not require an a priori known network structure to infer the logics. The proposed probabilistic logic is superior to the existing fuzzy logics and is more relevant to the biological contexts than the fuzzy logics. The performance of the LogicNet is superior to that of several Mutual Information-based and regression-based tools for reconstructing GRNs.

**Conclusions:**

The LogicNet reconstructs GRNs and logic functions without requiring prior knowledge of the network structure. Moreover, in another application, the LogicNet can be applied for logic function detection from the known regulatory genes-target interactions. We also conclude that computational modeling of the logical interactions among the regulatory genes significantly improves the GRN reconstruction accuracy.

## Background

The reconstruction of the gene regulatory networks (GRNs) is an important problem in molecular biology, which attempts to represent the causality of regulatory processes. The use of high-throughput microarray technologies to generate gene expression data has significantly facilitated network studies. The DREAM (the Dialogue for Reverse Engineering Assessments and Methods) program was initiated to encourage researchers to develop robust computational tools to infer GRNs from gene expression data [1].

The computational tools for the GRN inference can be classified into different categories. Abstract techniques such as the Principle Component Analysis (PCA) and Mutual Information (MI) [2–7] between genes are largely data-driven models in which the correlations among gene expression data are modelled. At the other extreme, differential equation-based models highly rely on prior knowledge about the network structure and the regulatory interactions. However, the temporal and spatial dynamics of each interaction can be captured by these models [8–10].

The knowledge-based models could rely on the prior information, e.g., reference regulatory networks documented in the databases, and then these reference networks are trimmed based on their consistencies with the gene expressions [11–13]. The prior knowledge is useful for the inference due to the noisy data in the -omics technology. A few differential equation-based and Bayesian models are proposed to reconstruct the GRNs from time-series microarrays, but they do not infer the logics among regulatory genes [14–17].

In the middle between the two extremes, there are Bayesian models, and logic-based models [18–24]. Logic-based models apply either a Boolean logic [20, 21, 25] or a multi-state logic [26–28] to study a priori-specified GRNs by using discretized gene expression data. While the normalized gene expression levels vary in the interval [0, 1], it is assumed in the Boolean networks that each gene is either expressed or not. Boolean logics apply a threshold on the interval [0, 1] to discretize the gene expression levels, resulting in the missing information. To overcome this weakness of the Boolean and the multi-state logics, the fuzzy logic models have been proposed to study the networks from the continuous gene expression data [19, 22]. However, the fuzzy and the multi-state logics study only a network with an a priori-specified structure and do not reconstruct it. Here, we introduce a new logic for continuous data, rather than binary data, called the probabilistic continuous (PC) logic, and accordingly, we propose a logic-based algorithm to reconstruct the GRNs from the continuous gene expression data. This new algorithm, called the LogicNet, is superior to the current logic-based models from several perspectives and has the following properties:

- The LogicNet relies on a new kind of logic applicable to continuous data, i.e., the PC logic, for modeling the cooperative, competitive and other types of logical interactions among genes. Regarding the reconstruction of the GRNs from the continuous gene expression data, the performance of the PC logic is superior to that of the fuzzy logic;
- Contrary to the current logic-based models, which can analyze only the GRNs with an a priori known structure, the LogicNet requires no prior information or hypothesis about the network structure;
- Using the continuous gene expression data in the interval [0, 1], the LogicNet reconstructs the GRN with directed and signed edges. Indeed, the LogicNet infers the underlying biochemical causalities of the regulatory interactions;
- The LogicNet infers the underlying logical relationships, e.g., the cooperative (AND, OR), competitive (XOR), and any other types of relationships, among the regulatory genes of a target gene.

Altogether, the main feature of the LogicNet is to improve the current models with the logic detection and not to defeat them in terms of accuracies. To study the regulatory effect of other genes on a target gene, the LogicNet computes the likelihood function for each possible set of regulatory genes with a specified logical interaction. In the LogicNet, the expression levels of the target gene belonging to the interval [0, 1] are intuitively supposed to follow a beta distribution. The parameters of this distribution depend on the type of the logical interaction of the regulatory genes. To prevent the model from over-fitting, the LogicNet applies the Bayesian Information Criterion (BIC) to force a balance between the quality of the fitting and the complexity of the interactions. The significance of the causal interactions is consequently modeled by using the Bayes Factor (BF).

## Results

The LogicNet performance is evaluated by using the simulated data from *Escherichia coli* (*E. coli*) and also data from the yeast GRNs of DREAM3 [1]. Also, the LogicNet performance is compared to several state-of-the-art tools, i.e., PCA-CMI [3], ARACNe [5], Genie3 [29], Narromi [4], CN [30], and GRNTE [31]. The performance is evaluated by using the true positive rate (TPR), false positive rate (FPR), positive predictive value (PPV), accuracy (ACC) and Matthews’s coefficient constant (MCC) defined as follows:

$$ \mathrm{TPR}=\mathrm{TP}/\left(\mathrm{TP}+\mathrm{FN}\right) $$

$$ \mathrm{FPR}=\mathrm{FP}/\left(\mathrm{FP}+\mathrm{TN}\right) $$

$$ \mathrm{PPV}=\mathrm{TP}/\left(\mathrm{TP}+\mathrm{FP}\right) $$

$$ \mathrm{ACC}=\left(\mathrm{TP}+\mathrm{TN}\right)/\left(\mathrm{TP}+\mathrm{FP}+\mathrm{TN}+\mathrm{FN}\right) $$

$$ MCC=\frac{TP\ast TN- FP\ast FN}{\sqrt{\left( TP+ FP\right)\left( TP+ FN\right)\left( TN+ FP\right)\left( TN+ FN\right)}} $$

where TP, FP, TN and FN are the numbers of the true positive, false positive, true negative, and false negative predictions, respectively. The LogicNet has no parameter by which we could calculate the receiver operating characteristic (ROC) curves and the area under the ROC curve (AUC). Therefore, the F-measure, which is the harmonic mean of the TPR and PPV, is used to compare the overall performance of the LogicNet with that of other tools. Although the LogicNet is compared to other tools for detecting undirected/directed network edges, it is also capable of detecting the underlying logic of the regulatory interactions. This capability is one advantage of the LogicNet for reconstructing the GRNs, and no other tool is currently capable of simultaneously detecting the directed network edges and the logic functions.

To evaluate the integrative performance of the LogicNet for the simultaneous detection of the directed edges and the logic functions, we apply a new measure in which we consider a TP if the regulatory genes and the active partition in the Venn diagram are both correctly predicted. In addition, we consider an FP if either the regulatory genes or the active partitions in the Venn diagram are predicted falsely. All other predictions are considered as FN (See the Methods section for more details).

### *E. coli* network with simulated logic functions

**Fig. 1:**
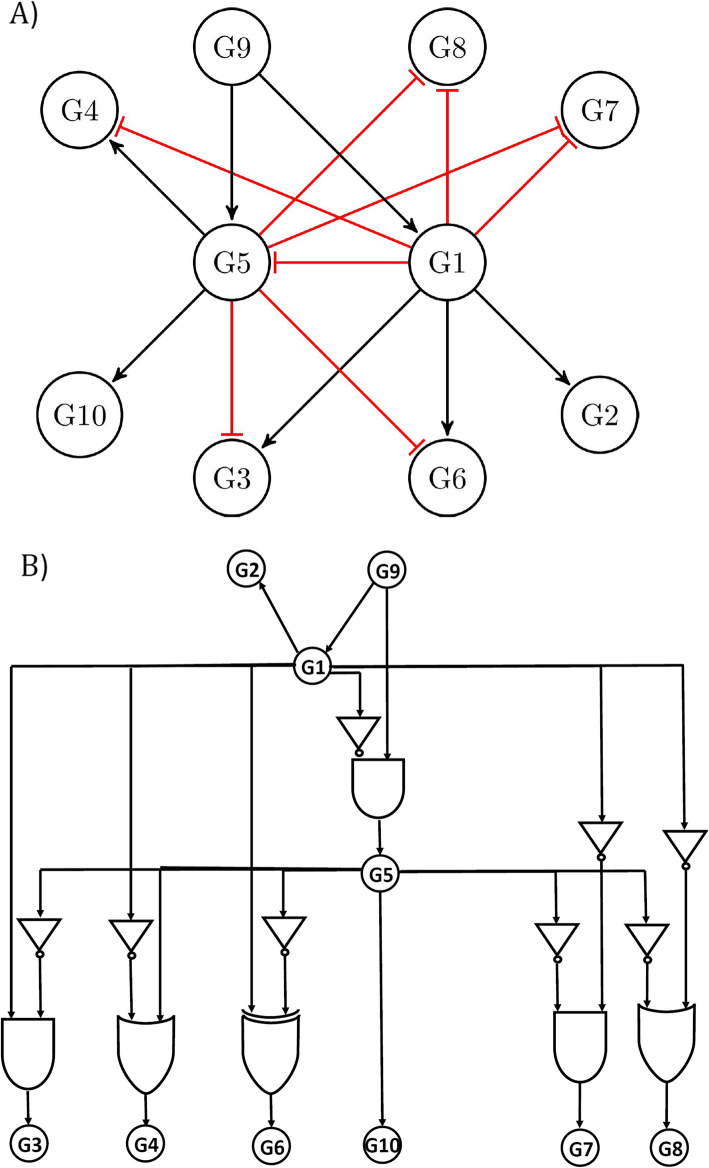
E. coli GRN and the simulated logic functions among the regulatory genes. **a** E. coli GRN from DREAM3 is shown. Activatory and inhibitory interactions are shown by the black and red edges, respectively. **b** E. coli GRN with simulated logic functions among the regulatory genes is shown

Figure 1 A shows the GRN of *E. coli* from the DREAM3 dataset in which the activatory and the inhibitory interactions are shown by the black and red edges, respectively. Since the logic functions among the regulatory genes are unknown, the *E. coli* logic functions are simulated with randomly assigned logics of types AND, OR, and XOR. Figure 1. B shows a possible logical network with simulated logics among the regulatory genes, constructed based on the *E. coli* network in Fig. 1. A. The gene expression samples are then simulated from this logical network, and the LogicNet is applied to predict the directed network and the logic functions. Table 1 shows the LogicNet performance separately for 10 and 50 gene expression samples and 100 repeats of the whole simulation study

**Table 1:** The LogicNet performance in predicting the GRNs and the logic functions, for 100 logic function simulations. The performance is evaluated at three levels, i.e., for undirected/directed networks and for directed logical networks in which the integrative detection of the directed edges and logic functions is evaluated

	Sample size	GRN	TPR	FPR	PPV	ACC	MCC	F-measure
PC-LogicNet	10	Undirected	0.48	0.05	0.82	0.79	0.51	0.61
		Directed	0.42	0.08	0.51	0.84	0.37	0.46
		Directed Logical	0.42	–	0.52	–	–	0.46
	50	Undirected	0.50	0.05	0.84	0.80	0.53	0.63
		Directed	0.44	0.08	0.53	0.84	0.39	0.48
		Directed Logical	0.43	–	0.53	–	–	0.47
Fuzzy-LogicNet	10	Undirected	0.43	0.05	0.81	0.77	0.46	0.56
		Directed	0.36	0.05	0.57	0.85	0.37	0.44
		Directed Logical	0.09	–	0.13	–	–	0.10
	50	Undirected	0.50	0.10	0.72	0.77	0.45	0.59
		Directed	0.43	0.07	0.55	0.85	0.40	0.48
		Directed Logical	0.08	–	0.10	–	–	0.09

As indicated in Table 1 for 10 samples, in detecting the undirected and directed GRN of *E. coli*, the PC-LogicNet reaches the F-measures of 0.61 and 0.46, respectively, which are superior to the performance of PCA-CMI [3], ARACNe [5], Genie3 [29], Narromi [4], CN [30], and GRNTE [31] (see Table 2 for comparisons).

**Table 2:** The LogicNet in comparison with PCA-CMI, ARACNe, Genie3, Narromi, CN, and GRNTE in reconstructing the undirected/directed *E. coli* network, using 10 gene expression samples and 100 repeats of the whole simulation study. Two types of logics, i.e., PC and fuzzy logics, are used separately for reconstructing the GRNs and logic functions in the LogicNet algorithm. Also, the value of *c* = *α* + *β* is set to 1000. The highest accuracies are indicated in boldface. Reported values for the TP, FP, TN, FN are the total of the corresponding values over 100 repeats of the whole simulation study

Method	TP	FP	TN	FN	TPR	FPR	PPV	ACC	MCC	F-measure
*Undirected E. coli* Network (the edge direction is not taken into account in calculating the performance)										
PC-LogicNet	724	157	2843	776	0.48	**0.05**	**0.82**	**0.79**	**0.51**	**0.61**
Fuzzy-LogicNet	640	155	2845	860	0.43	**0.05**	0.81	0.77	0.46	0.56
PCA-CMI-0.1	824	1974	1026	676	0.55	0.66	0.29	0.41	−0.11	0.38
PCA-CMI-0.05	940	2214	786	560	0.63	0.74	0.30	0.38	−0.11	0.40
ARACNe	160	140	2860	1340	0.11	0.05	0.53	0.67	0.11	0.18
GENIE3-FR-sqrt	213	228	2772	1287	0.14	0.08	0.48	0.66	0.10	0.22
GENIE3-FR-all	192	235	2765	1308	0.13	0.08	0.45	0.66	0.08	0.20
Narromi	490	829	2171	1010	0.33	0.28	0.37	0.59	0.05	0.35
CN	976	2297	703	524	**0.65**	0.77	0.30	0.37	−0.12	0.41
GRNTE	420	750	2241	1089	0.16	0.41	0.36	0.34	− 0.28	0.22
*Directed E. coli* Network										
PC-LogicNet	624	588	6912	876	**0.42**	0.08	0.51	0.84	**0.37**	**0.46**
Fuzzy-LogicNet	540	405	7095	960	0.36	0.05	**0.57**	**0.85**	**0.37**	0.44
ARACNe	120	180	7320	1380	0.08	**0.02**	0.40	0.83	0.12	0.13
GENIE3-FR-sqrt	155	445	7055	1345	0.10	0.06	0.26	0.80	0.07	0.15
GENIE3-FR-all	156	444	7056	1344	0.10	0.06	0.26	0.80	0.07	0.15
Narromi	275	1513	5987	1225	0.18	0.20	0.15	0.70	−0.02	0.17
CN	616	1369	6131	884	0.41	0.18	0.31	0.75	0.21	0.35
GRNTE	232	1210	3030	1128	0.04	0.59	0.13	0.15	−0.63	0.08

Table 1 also shows the integrative performance of the LogicNet in detecting both directed network and logic functions in *E. coli*. With this integrative measure, the PC- LogicNet reaches an F-measure of 0.46, which is significantly higher than its performance when using the fuzzy logic, i.e., 0.10. It should be noted that in achieving these results, the parameter *c,* i.e., *c* = *α* + *β*, is set to 1000 (See Methods). In Table 3, the sensitivity of the results is tested for other values of *c*, i.e., *c* = 500, 750, 1000 and 1250. As this table indicates, the results are not sensitive to the *c* values.

**Table 3:** The PC-LogicNet performance is evaluated for different values of *c* = *α* + *β*, i.e. c = 500, 750, 1000 and 1250. The PC-LogicNet is applied to reconstruct the directed network and logic functions among the regulatory genes in the *E. coli*, by using 10 gene expression samples and 100 repeats of the whole simulation study. Reported values for the TP, FP, TN, FN are the total of the corresponding values over 100 repeats of the whole simulation study

	Graph	TP	FP	TN	FN	TPR	FPR	PPV	ACC	MCC	F-measure
c = 500	Undirected ^a^	716	165	2835	784	0.48	0.06	0.81	0.79	0.50	0.60
	Directed ^b^	616	592	6908	884	0.41	0.08	0.51	0.84	0.36	0.45
	Directed logical ^c^	614	592	–	885	0.41	–	0.51	–	–	0.45
c = 750	Undirected	720	155	2845	780	0.48	0.05	0.82	0.79	0.51	0.61
	Directed	620	590	6910	880	0.41	0.08	0.51	0.84	0.37	0.46
	Directed logical	605	590	–	899	0.40	–	0.51	–	–	0.45
c = 1000	Undirected	724	157	2843	776	0.48	0.05	0.82	0.79	0.51	0.61
	Directed	624	588	6912	876	0.42	0.08	0.51	0.84	0.37	0.46
	Directed logical	625	588	–	868	0.42	–	0.52	–	–	0.46
c = 1250	Undirected	758	147	2853	742	0.51	0.05	0.84	0.80	0.54	0.63
	Directed	658	571	6929	842	0.44	0.08	0.54	0.84	0.39	0.48
	Directed logical	632	571	–	845	0.43	–	0.53	–	–	0.47

^a^ The edge direction is not taken into account in calculating the performance

^b^ The edge direction is taken into account in calculating the performance

^c^ The integrative performance of the LogicNet in reconstructing both the edge direction and logic function among regulatory genes is evaluated

### Yeast network real data

**Fig. 2:**
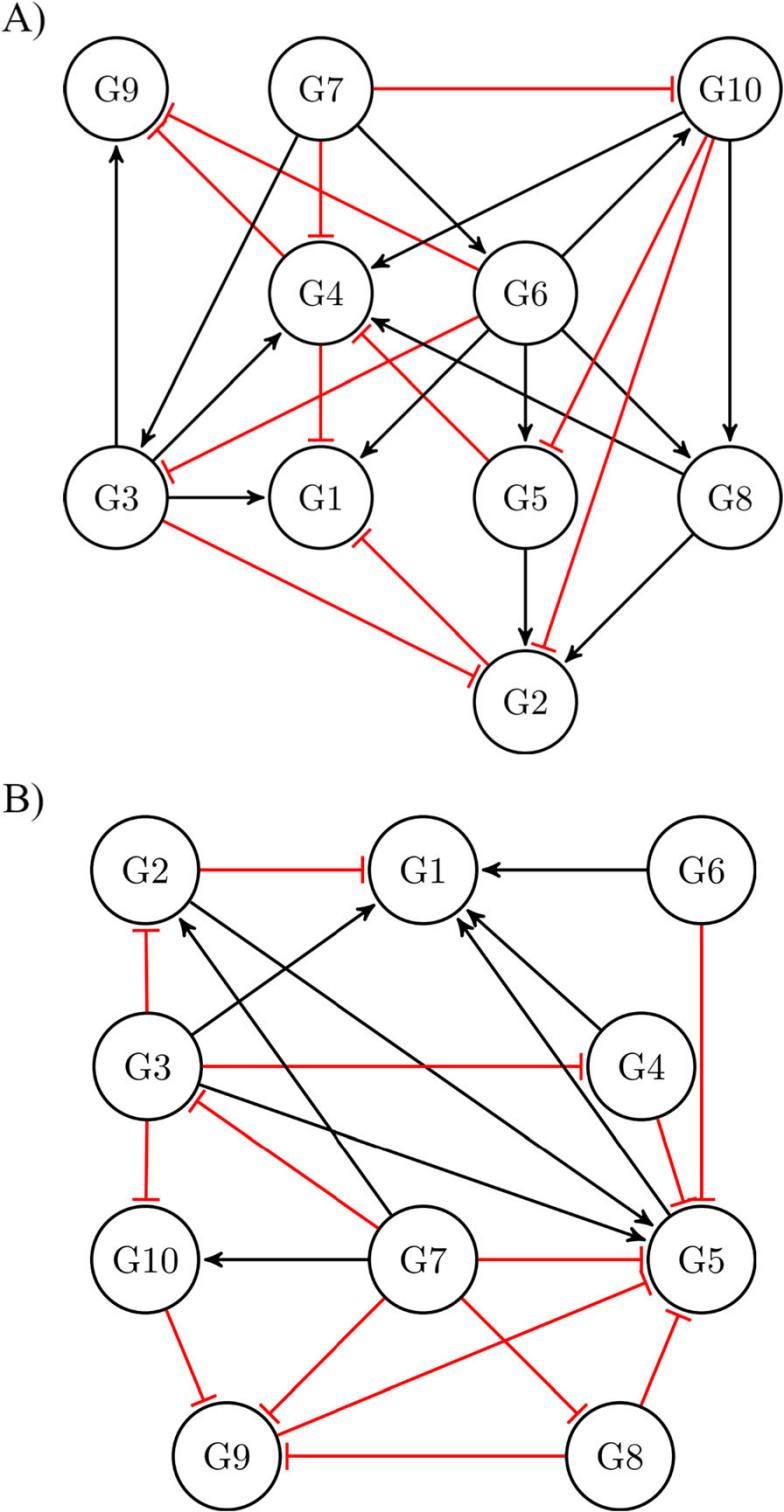
Yeast GRNs. **a** Yeast network Y2 with 10 nodes and 25 edges, **b** Yeast network Y3 with 10 nodes and 22 edges, as parts of the DREAM3 dataset

Figure 2 shows two yeast GRNs, i.e., Y2 and Y3, in which activatory and inhibitory interactions are respectively shown by the black and red edges. The microarray gene expression data of these networks are downloaded from the DREAM3 dataset, and the LogicNet is applied for reconstructing the networks. See Table 4 for the predicted edges and logics.

**Table 4:** The predicted regulators and logic functions among these regulatory genes in *Y2* and *Y3* networks, with LogicNet

Gene	Predicted Regulator/Logic Function
Y2 network	
*G*_1_	${G}_6{G}_8{G}_9\bigvee \overline{G_6}{G}_8{G}_9\bigvee {G}_6{G}_8\overline{G_9}\bigvee \overline{G_6}{G}_8\ \overline{G_9}\bigvee {G}_6\overline{G_8}\overline{G_9}$
*G*_2_	$\overline{G_1}{G}_5{G}_8\bigvee {G}_1\overline{G_5}{G}_8\bigvee {G}_1{G}_5\overline{G_8}\bigvee \overline{G_1}{G}_5\overline{G_8}\bigvee {G}_1\overline{G_5}\ \overline{G_8}$
*G*_3_	${G}_4{G}_5{G}_9\bigvee {G}_4\overline{G_5}\overline{G_9}$
*G*_4_	${G}_5\overline{G_7}{G}_{10}\bigvee \overline{G_5}\overline{G_7}\overline{G_{10}}$
*G*_5_	$\overline{G_3}\overline{G_8}{G}_{10}\bigvee \overline{G_3}{G}_8\overline{G_{10}}\bigvee \overline{G_3}\overline{G_8}\overline{G_{10}}$
*G*_6_	${G}_1{G}_2{G}_7\bigvee \overline{G_1}{G}_2{G}_7\bigvee {G}_1\overline{G_2}{G}_7\bigvee \overline{G_1}\overline{G_2}{G}_7\bigvee \overline{G_1}{G}_2\overline{G_7}$
*G*_7_	$\overline{G_4}{G}_6{G}_9\bigvee {G}_4\overline{G_6}{G}_9\bigvee {G}_4{G}_6\overline{G_9}\bigvee \overline{G_4}{G}_6\overline{G_9}\bigvee {G}_4\overline{G_6}\ \overline{G_9}\bigvee \overline{G_4}\overline{G_6}\overline{G_9}$
*G*_8_	${G}_2\overline{G_3}{G}_9\bigvee {G}_2{G}_3\overline{G_9}\bigvee \overline{G_2}\overline{G_3}\overline{G_9}$
*G*_9_	${G}_2{G}_6{G}_8\bigvee \overline{G_2}{G}_6{G}_8\bigvee \overline{G_2}\overline{G_6}\overline{G_8}$
*G*_10_	$\overline{G_6}{G}_8{G}_9$
Y3 network	
*G*_1_	${G}_2{G}_4\overline{G_6}\bigvee \overline{G_2}\overline{G_4}{G}_6$
*G*_2_	$\overline{G_3}{G}_7{G}_8\bigvee {G}_3{G}_7\overline{G_8}\bigvee \overline{G_3}{G}_7\overline{G_8}\bigvee \overline{G_3}\overline{G_7}\overline{G_8}$
*G*_3_	${G}_1{G}_4{G}_5\bigvee \overline{G_1}\overline{G_4}{G}_5$
*G*_4_	${G}_3{G}_5{G}_{10}\bigvee {G}_3\overline{G_5}{G}_{10}\bigvee \overline{G_3}\overline{G_5}{G}_{10}\bigvee \overline{G_3}{G}_5\overline{G_{10}}\bigvee \overline{G_3}\overline{G_5}\overline{G_{10}}$
*G*_5_	$\overline{G_4}\overline{G_7}{G}_8\bigvee \overline{G_4}{G}_7\overline{G_8}\bigvee \overline{G_4}\overline{G_7}\overline{G_8}$
*G*_6_	${G}_2{G}_5{G}_9\bigvee \overline{G_2}{G}_5{G}_9\bigvee {G}_2\overline{G_5}{G}_9\bigvee {G}_2{G}_5\overline{G_9}\bigvee \overline{G_2}\overline{G_5}{G}_9\bigvee \overline{G_2}{G}_5\overline{G_9}\bigvee {G}_2\overline{G_5}\ \overline{G_9}$
*G*_7_	$\overline{G_3}{G}_6{G}_8\bigvee {G}_3\overline{G_6}{G}_8\bigvee \overline{G_3}{G}_6\overline{G_8}\bigvee {G}_3\overline{G_6}\ \overline{G_8}\bigvee \overline{G_3}\overline{G_6}\overline{G_8}$
*G*_8_	*–*
*G*_9_	$\overline{G_1}{G}_5{G}_6\bigvee {G}_1{G}_5\overline{G_6}\bigvee {G}_1\overline{G_5}\ \overline{G_6}$
*G*_10_	$\overline{G_3}{G}_7{G}_8\bigvee \overline{G_3}{G}_7\overline{G_8}\bigvee \overline{G_3}\overline{G_7}\overline{G_8}$

In Table 5, the performance of the LogicNet in reconstructing the undirected yeast networks is compared with that of other tools, (see Table 6 for the results of predicting the directed networks). As Table 5 illustrates, the LogicNet outperforms the other tools in reconstructing the undirected networks of Y2 and Y3, with an F-measure of 0.60 and 0.74, respectively. Moreover, as shown in Tables 5 and 6, the performance of the PC logic is superior to that of the fuzzy logic, in the majority of cases. These results indicate that the PC logic is more effective and relevant to the biological processes in logic function modeling than the fuzzy logic.

**Table 5:** The LogicNet in comparison with PCA-CMI, ARACNe, Genie3, Narromi, CN, and GRNTE in reconstructing the undirected yeast networks (the edge direction is not taken into account in calculating the performance). Yeast networks Y2 and Y3 are reconstructed by using 10 gene expression samples from the DREAM3 dataset. Two types of logics, i.e., the PC and the fuzzy logics, are used separately for reconstructing the GRNs and detecting the logic functions in the LogicNet algorithm. The value of *c* = *α* + *β* is set to 1000. The highest accuracies are indicated in boldface

Method	TP	FP	TN	FN	TPR	FPR	PPV	ACC	MCC	F-measure
Yeast Network Y2										
PC-LogicNet	14	10	10	11	**0.56**	0.50	0.58	0.53	0.06	0.57
Fuzzy-LogicNet	14	8	12	11	**0.56**	0.40	0.64	**0.58**	0.16	**0.60**
PCA-CMI-0.1	5	1	19	20	0.20	0.05	0.83	0.53	0.22	0.32
PCA-CMI-0.05	5	2	18	20	0.20	0.10	0.71	0.51	0.14	0.31
ARACNe	1	0	20	24	0.04	**0.00**	**1.00**	0.47	0.13	0.08
GENIE3-FR-sqrt	5	1	19	20	0.20	0.05	0.83	0.53	0.22	0.32
GENIE3-FR-all	3	3	17	22	0.12	0.15	0.50	0.44	−0.04	0.19
Narromi	8	2	18	17	0.32	0.10	0.80	**0.58**	**0.26**	0.46
CN	8	5	15	17	0.32	0.25	0.62	0.51	0.08	0.42
GRNTE	14	9	11	11	**0.56**	0.45	0.61	0.56	0.11	0.58
Yeast Network Y3										
PC-LogicNet	17	7	16	5	**0.77**	0.30	0.71	0.73	0.47	**0.74**
Fuzzy-LogicNet	14	8	15	8	0.64	0.35	0.64	0.64	0.29	0.64
PCA-CMI-0.1	14	2	21	8	0.64	0.09	0.88	**0.78**	**0.57**	**0.74**
PCA-CMI-0.05	15	6	17	7	0.68	0.26	0.71	0.71	0.42	0.70
ARACNe	3	0	23	19	0.14	**0.00**	**1.00**	0.58	0.27	0.24
GENIE3-FR-sqrt	3	1	22	19	0.14	0.04	0.75	0.56	0.16	0.23
GENIE3-FR-all	3	2	21	19	0.14	0.09	0.60	0.53	0.08	0.22
Narromi	6	5	18	16	0.27	0.22	0.55	0.53	0.06	0.36
CN	17	7	16	5	0.77	0.30	0.71	0.73	0.47	**0.74**
GRNTE	10	7	16	12	0.45	0.30	0.59	0.58	0.15	0.51

**Table 6:** The LogicNet in comparison with ARACNe, Genie3, Narromi, CN, and GRNTE in reconstructing the directed yeast networks. Two Yeast networks, i.e., Y2 and Y3 with 10 genes and 25 edges (Y2)/22 edges (Y3), are reconstructed by the LogicNet by using 10 gene expression samples from the DREAM3 dataset

Method	TP	FP	TN	FN	TPR	FPR	PPV	ACC	MCC	F-measure
Yeast Network Y2										
PC-LogicNet	10	20	45	15	0.40	0.31	0.33	0.61	0.09	0.36
Fuzzy-LogicNet	8	18	47	17	0.32	0.28	0.31	0.61	0.04	0.31
ARACNe	0	1	64	25	0.00	**0.02**	0.00	0.71	−0.07	–
GENIE3-FR-sqrt	1	5	60	24	0.04	0.08	0.17	0.68	−0.07	0.06
GENIE3-FR-all	1	5	60	24	0.04	0.08	0.17	0.68	−0.07	0.06
Narromi	6	5	60	19	0.24	0.08	**0.55**	**0.73**	**0.22**	0.33
CN	1	5	60	24	0.04	0.08	0.17	0.68	−0.07	0.06
GRNTE	12	17	48	13	**0.48**	0.26	0.41	0.67	0.21	**0.44**
Yeast Network Y3										
PC-LogicNet	11	16	52	11	**0.50**	0.24	0.41	0.70	**0.25**	**0.45**
Fuzzy-LogicNet	8	19	49	14	0.36	0.28	0.30	0.63	0.08	0.33
ARACNe	1	2	66	21	0.05	**0.03**	0.33	0.74	0.04	0.08
GENIE3-FR-sqrt	2	2	66	20	0.09	**0.03**	**0.50**	**0.76**	0.13	0.15
GENIE3-FR-all	1	5	63	21	0.05	0.07	0.17	0.71	−0.05	0.07
Narromi	5	7	61	17	0.23	0.10	0.42	0.73	0.16	0.29
CN	6	11	57	16	0.27	0.16	0.35	0.70	0.12	0.31
GRNTE	7	15	53	15	0.32	0.22	0.32	0.67	0.10	0.32

It should be emphasized that PCA-CMI [3], ARACNe [5], Genie3 [29], Narromi [4], CMI2NI [2], and CN [30] are threshold dependent. These thresholds, e.g., on mutual information between two genes, determine the significance of the regulatory interactions. As these thresholds are user-dependent, and there is no a priori information to determine them, many of the current tools are limited by their dependency on a threshold. However, in the LogicNet, due to the large difference in the likelihoods of the target’s gene expression level under a biologically significant logic and a random logic, we can always decisively infer the significant logic functions with a BF > 100.

### Application to the logic function detection

The LogicNet can also be applied to infer the logic functions among the regulatory genes, in the networks with a known structure. For this purpose, we used the previously identified gene regulation in the yeast with 176 Regulatory Factors (RFs) and their target genes [32, 33]. The number of target genes with 1, 2 and 3 RFs are, respectively, 1472, 1013 and 653. To infer the logic function among these regulatory genes, the LogicNet is fed with three well-studied yeast cell-cycle datasets [34, 35]: 1) the alpha-factor time course with 16 time points (0, 7′, …, 119′); 2) cdc15 time course with 25 time points (10′, 30′, …, 290′); and 3) cdc28 time course with 17 time points (0, 10′, …, 160′) for the gene expression samples. After combining all three datasets (5581 genes and 58 time points), the gene expressions for each time point are converted into the interval [0, 1].

**Fig. 3:**
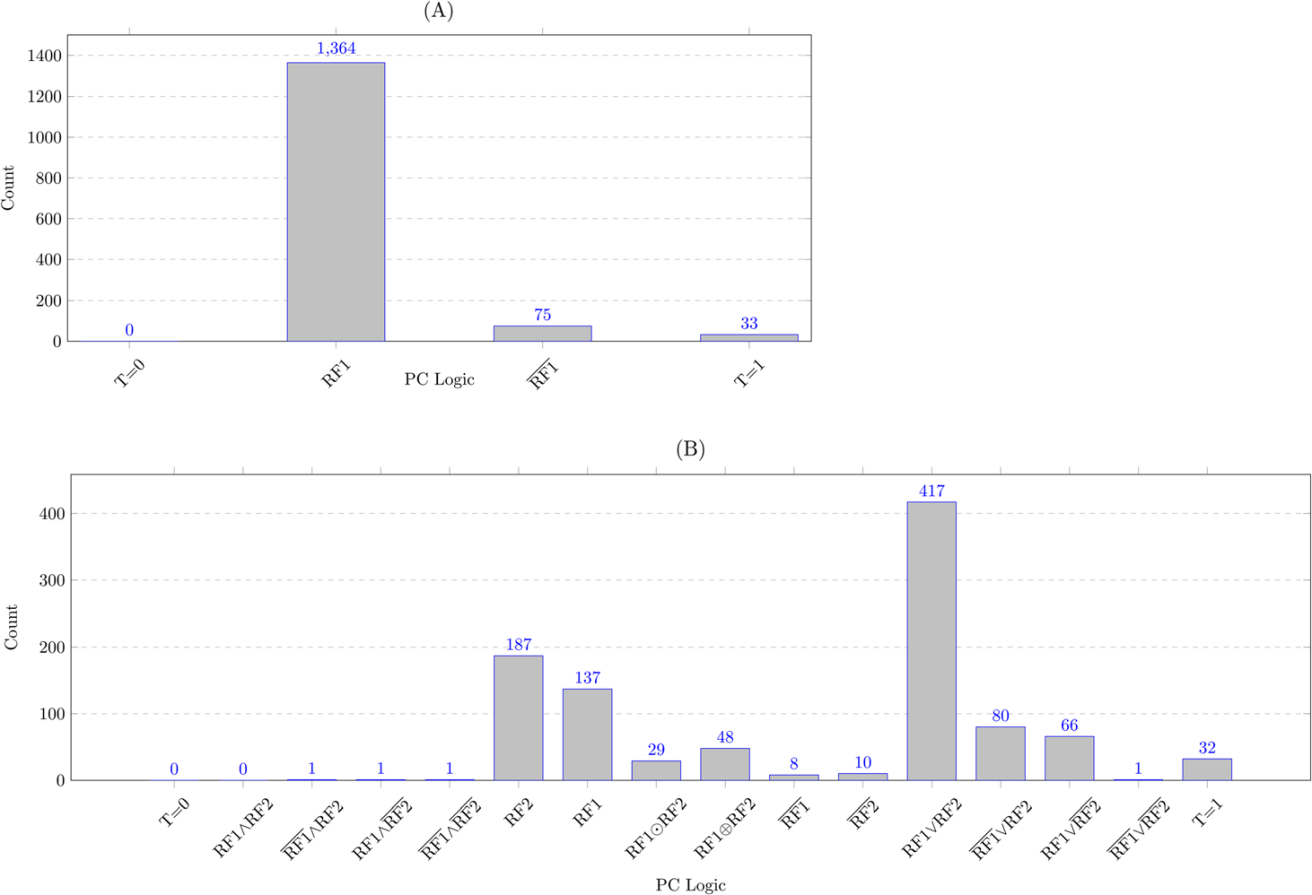
The number of PC logic functions which are inferred by the LogicNet in the yeast. **a** The target genes are regulated by one RF. **b** The target genes are regulated by two RFs

For target genes with one RF, we used the LogicNet to characterize the RF1-target logics during the yeast cell cycle. As depicted in Fig. 3. A, we found 1364 RF-target logics of type Target = RF1 and 75 logics of type $\mathrm{Target}=\overline{\mathrm{RF}1}$. The other 33 RF-target logics were of type Target = 1. See Supplementary File 1 for the gene names with RF-target interaction and the corresponding logic function.

For the target genes with two RFs, we used the LogicNet to characterize the RF1-RF2-target logics by computing the likelihood values for the 16 possible logic functions among two RFs, as shown in Table 7. As depicted in Fig. 3. B, logic functions “Target = RF1VRF2” (i.e., OR logic function), “Target = RF2” and “Target = RF1” are more frequent than the other logic functions for characterizing RF1-RF2-target logics. The OR logic for the RF1-RF2-target interaction indicates that either RF1 or RF2 is enough to activate the expression of their target genes. Also, the non-cooperative logic functions such as “Target = RF2” and “Target = RF1” indicate that only one RF (the dominant RF) controls the target regulation. See Supplementary File 1 for the gene names with RF1-RF2-target interaction and the corresponding logic function. We also used the LogicNet to characterize the RF1-RF2-RF3-target logics by computing the likelihood values for the 256 possible logic functions among three RFs (see Supplementary File 1 for the result).

**Table 7:** 16 possible PC logic functions between two genes *G*_1_ and *G*_2_, which regulate the target. The ∪ sign stands for the union of the sets, and ∨, ∧ , ⊕ , and ⨀ stand for the OR, AND, XOR, and XNOR PC logics between *G*_1_ and *G*_2_

*i*	*i*_3_	*i*_2_	*i*_1_	*i*_0_	*f*_*i*_(*G*_1_, *G*_2_)	*Output*
0	0	0	0	0	0	0
1	0	0	0	1	$\overline{G_1}\overline{G_2}=\overline{G_1}\wedge \overline{G_2}$	$\left(1-{\mathit{\exp}}_{G_1}\right)\ast \left(1-{\mathit{\exp}}_{G_2}\right)$
2	0	0	1	0	$\overline{G_1}{G}_2=\overline{G_1}\wedge {G}_2$	${\mathit{\exp}}_{G_2}-{\mathit{\exp}}_{G_1}\ast {\mathit{\exp}}_{G_2}$
3	0	0	1	1	$\overline{G_1}{G}_2\cup \overline{G_1}\overline{G_2}=\overline{G_1}$	$1-{\mathit{\exp}}_{G_1}$
4	0	1	0	0	${G}_1\overline{G_2}={G}_1\wedge \overline{G_2}$	${\mathit{\exp}}_{G_1}-{\mathit{\exp}}_{G_1}\ast {\mathit{\exp}}_{G_2}$
5	0	1	0	1	${G}_1\overline{G_2}\cup \overline{G_1}\overline{G_2}=\overline{G_2}$	$1-{\mathit{\exp}}_{G_2}$
6	0	1	1	0	${G}_1\overline{G_2}\cup \overline{G_1}{G}_2={G}_1\bigoplus {G}_2$	${\mathit{\exp}}_{G_1}+{\mathit{\exp}}_{G_2}-2\ {\mathit{\exp}}_{G_1}\ast {\mathit{\exp}}_{G_2}$
7	0	1	1	1	${G}_1\overline{G_2}\cup \overline{G_1}{G}_2\cup \overline{G_1}\overline{G_2}=\overline{G_1}\vee \overline{G_2}$	$1-{\mathit{\exp}}_{G_1}\ast {\mathit{\exp}}_{G_2}$
8	1	0	0	0	*G*_1_*G*_2_ = *G*_1_ ∧ *G*_2_	${\mathit{\exp}}_{G_1}\ast {\mathit{\exp}}_{G_2}$
9	1	0	0	1	${G}_1{G}_2\cup \overline{G_1}\overline{G_2}={G}_1\bigodot {G}_2$	$1-{\mathit{\exp}}_{G_1}-{\mathit{\exp}}_{G_2}+2\ {\mathit{\exp}}_{G_1}\ast {\mathit{\exp}}_B$
10	1	0	1	0	${G}_1{G}_2\cup \overline{G_1}{G}_2={G}_2$	${\mathit{\exp}}_{G_2}$
11	1	0	1	1	${G}_1{G}_2\cup \overline{G_1}{G}_2\cup \overline{G_1}\overline{G_2}=\overline{G_1}\vee {G}_2$	$1-{\mathit{\exp}}_{G_1}+{\mathit{\exp}}_{G_1}\ast {\mathit{\exp}}_{G_2}$
12	1	1	0	0	${G}_1{G}_2\cup {G}_1\overline{G_2}={G}_1$	${\mathit{\exp}}_{G_1}$
13	1	1	0	1	${G}_1{G}_2\cup {G}_1\overline{G_2}\cup \overline{G_1}\overline{G_2}={G}_1\vee \overline{G_2}$	$1-{\mathit{\exp}}_{G_2}+{\mathit{\exp}}_{G_1}\ast {\mathit{\exp}}_{G_2}$
14	1	1	1	0	${G}_1{G}_2\cup {G}_1\overline{G_2}\cup \overline{G_1}{G}_2={G}_1\vee {G}_2$	${\mathit{\exp}}_{G_1}+{\mathit{\exp}}_{G_2}-{\mathit{\exp}}_{G_1}\ast {\mathit{\exp}}_{G_2}$
15	1	1	1	1	${G}_1{G}_2\cup {G}_1\overline{G_2}\cup \overline{G_1}{G}_2\cup \overline{G_1}\overline{G_2}=1$	1

As in previous studies [36], we used RF knockout experiments in the yeast to validate the logic functions which are inferred by the LogicNet. These RF knockout experiments measure the gene expression fold changes, after deleting each RF [37, 38]. If the target is cooperatively regulated by two RFs, e.g., in “Target = RF1VRF2” (OR logic), then it is most likely that the knockout of either RF decreases the target gene expressions. In 412 logic functions “Target = RF1VRF2”, which are inferred by the LogicNet, deleting either RF1 or RF2 decreases the target gene expression by a factor of − 0.016 and − 0.157 in the logarithm scale. For the non-cooperative logic functions, e.g., “Target = RF2”, we found that deleting the dominant RF, i.e., RF2 downregulates the target gene expression more than the removal of RF1. Indeed, in logic function “Target = RF2”, deleting RF1 or RF2 decreases the target gene expression on average by a factor of − 0.022 and − 0.086, respectively, with a standard deviation of 0.37 and 0.34.

### Application to RNA-Seq data

LogicNet is also applied to infer GRNs in the early embryonic development data (oocyte to E4.25 blastocyst stages) [39], from single-cell transcriptome sequencing of 48 genes. As described in the original study [39], raw Ct data are first subtracted by the detection limit of 28 and further normalized on a cell-wise basis by subtracting the mean expression of housekeeping genes Actb and Gapdh.

GRNs are then reconstructed for two overlapping subsets of data from 46 genes, i.e., excluding the housekeeping genes which are used for data normalization. The early subset of data includes the cells from oocyte up to 32-cell E3.5 blastocyst stages and the late subset includes the cells from 16-cell morula to 64-cell E4.25 blastocyst stages.

**Fig. 4:**
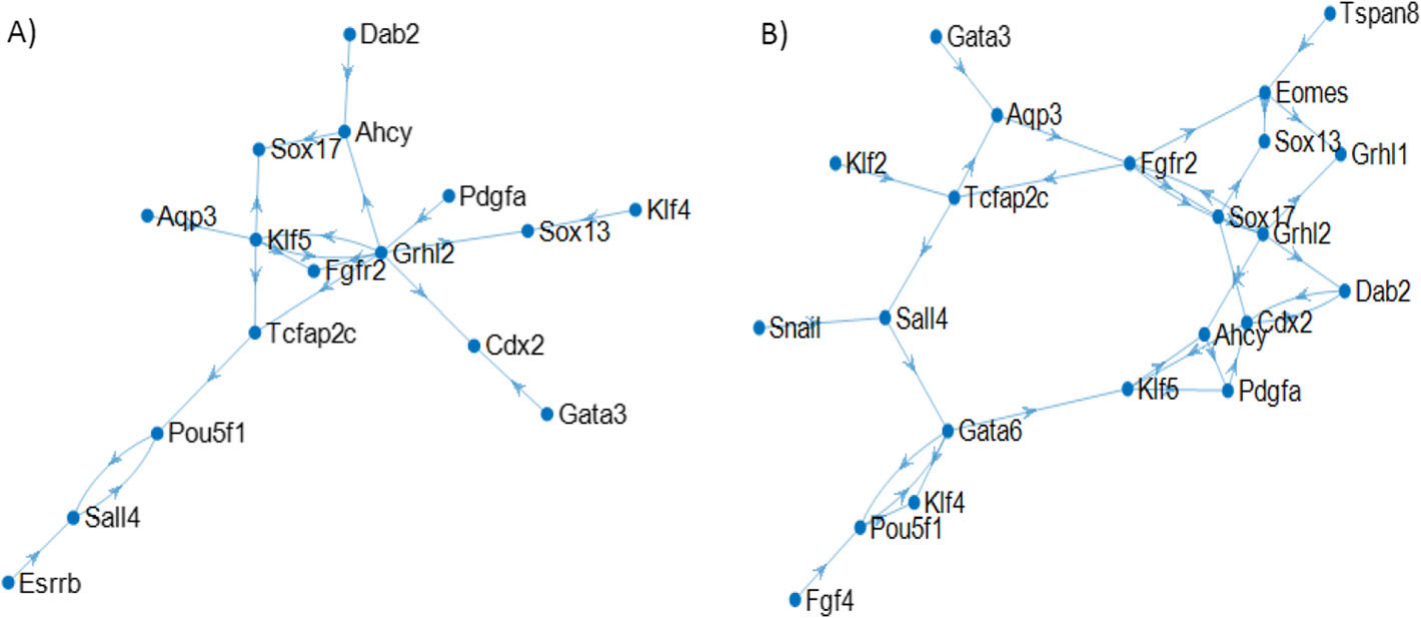
**a** For cells from oocyte up to 32-cell E3.5 blastocyst stages. **b** For cells from 16-cell morula to 64-cell E4.25 blastocyst stages

Inferred GRNs using LogicNet are depicted in panels A and B of Fig. 4, respectively for the early and late subsets of cells. As shown in this Figure, GRN for cells from 16-cell morula to 64-cell E4.25 blastocyst stages is more complex than GRN for the early subset of cells. However, in both networks, Grhl2 has an important role as a hub.

### The LogicNet complexity

To calculate the time complexity of the LogicNet, consider N genes in the network and a sample of *n* gene expression vectors. For each gene as a target and logic functions including up to k regulatory nodes, we have ${2}^2\left(\genfrac{}{}{0pt}{}{N-1}{1}\right)+{2}^{2^2}\left(\genfrac{}{}{0pt}{}{N-1}{2}\right)+\dots +{2}^{2^{\mathrm{k}}}\left(\genfrac{}{}{0pt}{}{N-1}{k}\right)$ possible logic functions in the model. Then, having N genes, each considered as a target at a time and a sample size of *n*, we reach a complexity of $O\left(n{2}^{2^{\mathrm{k}}}{N}^{k+1}\right)$ for the number of calculations in the model.

## Discussion

The PC-LogicNet achieves a considerably higher F-measure than the Fuzzy-LogicNet. This result indicates that the PC logic is more relevant and effective in modeling regulatory gene interactions. Therefore, future studies can benefit from this PC logic in reconstructing the GRNs and detecting the logic functions. Moreover, compared to the previous logic-based models, the LogicNet does not rely on a priori known network structure to infer the logic functions. However, as described in the results section, the LogicNet can be applied for the logic function detection from the known regulatory genes-target interactions.

Moreover, since the parameters of the beta distribution are estimated separately for each sample, the LogicNet can model the gene expression data that follow a multi-modal distribution. This capability is a major advantage of the LogicNet over many existing tools, which have difficulties in modeling the multi-modal gene expression data.

R package of the LogicNet is available at https://github.com/CompBioIPM/LogicNet. Yeast and E.coli data sets, which were used in this study, are also available on this webpage. Parallel programming of the LogicNet algorithm reduces its running time considerably. For a GRN of 10 nodes and 10 gene expression samples, it takes 275 s to run the LogicNet on a 64-bit operating system with an Intel(R) Core (TM) i7-4710HQ CPU @ 3.50 GHz processor and 16 GB RAM.

## Conclusion

The LogicNet performance is superior to that of the MI-based and regression-based tools. The low performance of these tools is, to some extent, associated with ignoring the logic function among the regulatory genes. Indeed, compared to the other tools, logic-based models are more accurate for reconstructing the GRNs and more useful for detecting the logic functions, two important problems in biology.

## Methods

The LogicNet was developed to infer the existing regulatory interactions of a target gene *T* and to determine the corresponding logic behind these interactions. The values of the expression level of each gene are normalized into the interval [0, 1]. In the LogicNet, these expression levels are supposed to be the samples of a beta distribution. In this context, the expression level expresses the probability of being an active gene. In other words, an expression level value close to zero indicates a high probability of being off. Accordingly, a regulatory gene with a higher level of activity is more probable to influence other genes. Furthermore, it is assumed that the expression levels of *T* are the outputs of a continuous logic function whose inputs are the gene expression level of the regulatory genes of *T*. Hence, each logic function provides an estimate of the expression level of *T*, or, similarly, an estimate of the probability of the activity of *T*. We call this function a probabilistic continuous (PC) logic function.

### PC logic function

**Fig. 5:**
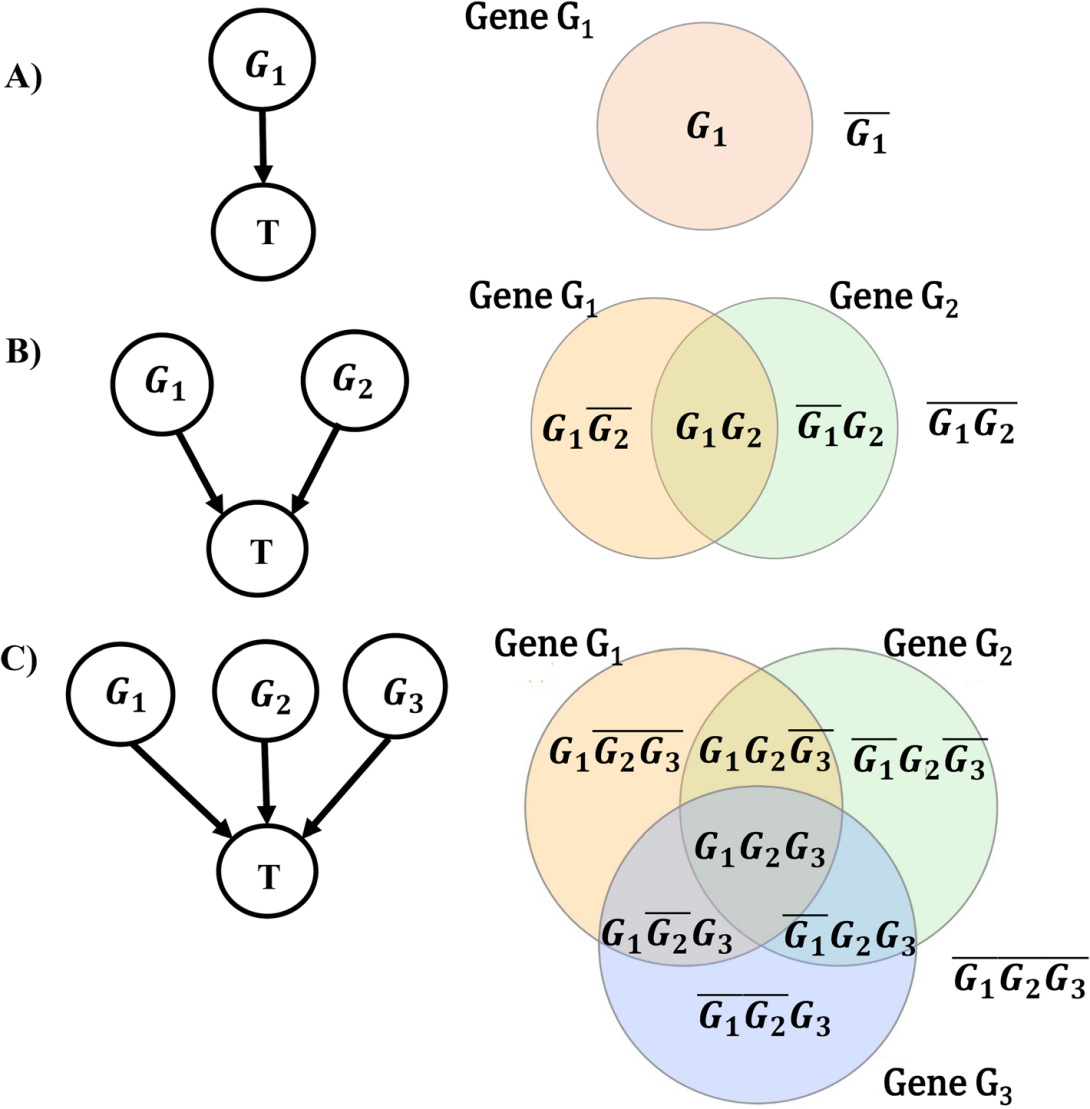
Venn diagram partitions representing different interactions among the regulatory genes influencing the target *T*. Each partition either exists or does not exist in the corresponding *f*_*i*_(*G*_1_, *G*_2_, …, *G*_*k*_) of the logic function. **a***G*_1 _regulates *T*. Each partition, i.e., *G*_1_ and $\overline{G_1}$ of the Venn diagram, is possibly on or off in *f*_*i*_(*G*_1_). **b** Both genes *G*_1_ and *G*_2_ regulate T. The Venn diagram is partitioned into 4 disjoint regions; each is potentially on or off in *f*_*i*_(*G*_1_, *G*_2_). **c** Genes *G*_1_, *G*_2_ and *G*_3_ regulate T. The Venn diagram is partitioned into 8 disjoint regions; each is potentially on or off in *f*_*i*_(*G*_1_, *G*_2_, *G*_3_)

Consider *k* genes *G*_1_, *G*_2_, …, *G*_*k*_ regulating the target gene *T*. Each gene can have an activatory or inhibitory effect on *T*, denoted by *G*_*i*_ and ${\overline{G}}_i$, respectively. Hence, there are 2^*k*^ different combinations of the activatory and inhibitory effects of all regulators, e.g.; for *k* = 1 we have *G*_1_ and $\overline{G_1}$ and for *k* = 2 we have 4 different combinations of *G*_1_*G*_2_, ${G}_1{\overline{G}}_2$, ${\overline{G}}_1{G}_2$, and ${\overline{G}}_1{\overline{G}}_2$. These activatory/inhibitory combinations can be associated with partitions in the Venn diagram of the set of *k* regulatory genes (Fig. 5). Now for *k* = 1, 2, and 3, and for the regulatory genes *G*_1_, *G*_2_, and *G*_3_, we use Venn diagram partitions and define PC logic functions as follows:

1 $$ {f}_i\left({G}_1\right)={i}_1{G}_1\cup {i}_0\overline{G_1} $$

2 $$ {f}_i\left({G}_1,{G}_2\right)={i}_3{G}_1{G}_2\cup {i}_2{G}_1\overline{G_2}\cup {i}_1\overline{G_1}{G}_2\cup {i}_0\overline{G_1}\overline{G_2} $$

3 $$ {f}_i\left({G}_1,{G}_2,{G}_3\right)={i}_7{G}_1{G}_2{G}_3\cup {i}_6\overline{G_1}{G}_2{G}_3\cup {i}_5{G}_1\overline{G_2}{G}_3\cup {i}_4{G}_1{G}_2\overline{G_3}\cup {i}_3\overline{G_1}\overline{G_2}{G}_3\cup {i}_2\overline{G_1}{G}_2\overline{G_3}\cup {i}_1{G}_1\overline{G_2}\overline{G_3}\cup {i}_0\overline{G_1}\overline{G_2}\overline{G_3}, $$

where ∪ stands for the union of two sets, and ${\left({i}_{2^k-1},\dots, {i}_2,{i}_1,{i}_0\right)}_2$ denotes the binary representation of the PC logic function index *i*. Indeed, the coefficient of each partition in *f*_*i*_(*G*_1_, *G*_2_, …, *G*_*k*_) could be 0 or 1, indicating the presence of the corresponding activatory/inhibitory combination in *f*_*i*_(*G*_1_, *G*_2_, …, *G*_*k*_), for more details on notations see [40]. Moreover, binary variables ${\left({i}_{2^k-1},\dots, {i}_2,{i}_1,{i}_0\right)}_2$ in the PC logic function provide a systematic way to generate different logics and these random variables have to be estimated in our maximum likelihood approach, in the next subsections. In general, according to the ${\left({i}_{2^k-1},\dots, {i}_2,{i}_1,{i}_0\right)}_2$, there are ${2}^{2^k}$ different PC logic functions *f*_*i*_(*G*_1_, *G*_2_, …, *G*_*k*_) for *k* regulatory genes, where $0\le i<{2}^{2^k}$.

**Fig. 6:**
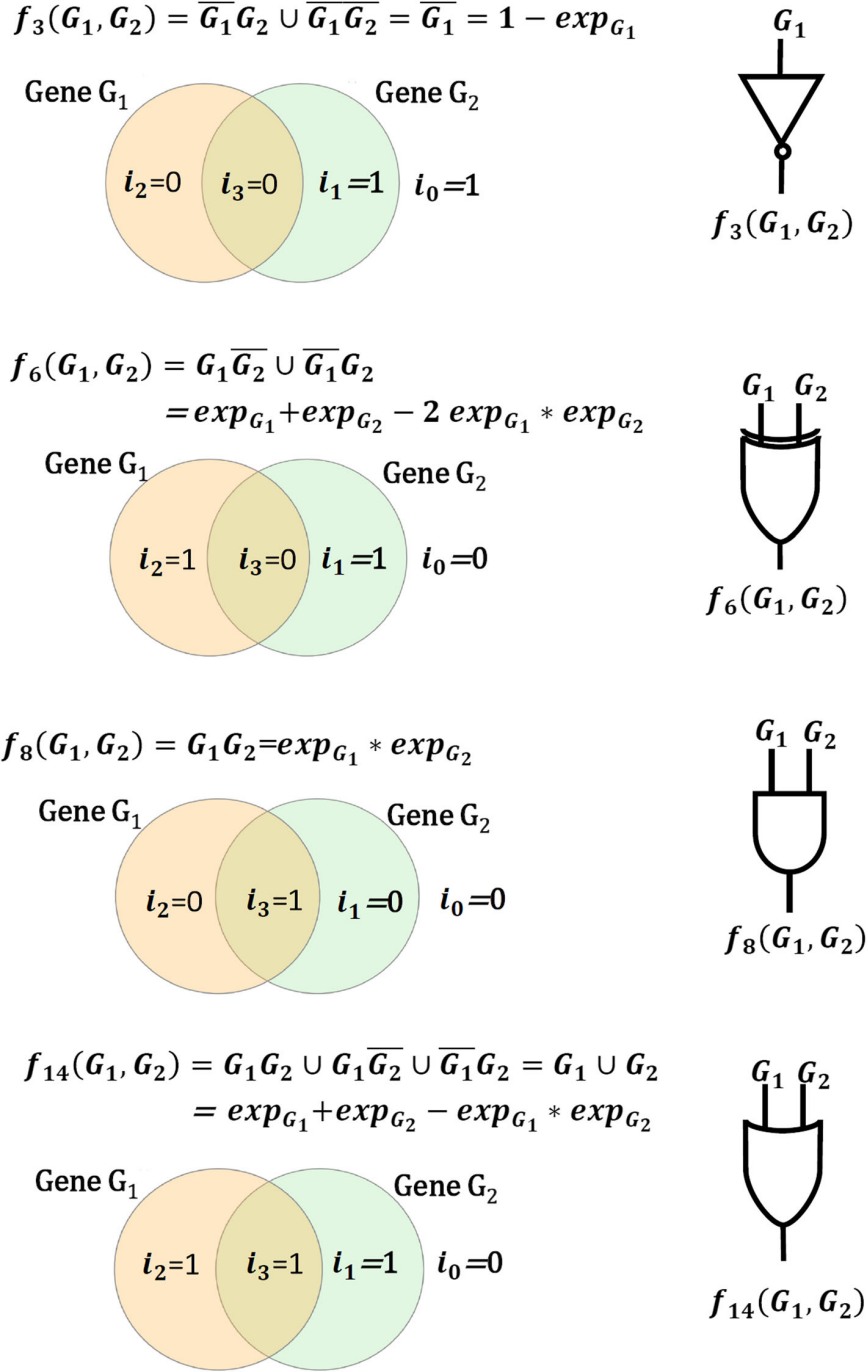
Participating activatory/inhibitory partitions in the Venn diagram for logic functions f_3_, f_6_, f_8_, and f_14_. The indexes i_0_, i_1_, i_2_ and i_3_ indicate if the corresponding partition exists in f_i_(*G*_1_, *G*_2_), between genes *G*_1_ and *G*_2_

The occurrence of each partition in the PC logic function results in the expression of the target gene *T*. Each partition represents the AND logic between the genes, e.g., *f*_8_(*G*_1_, *G*_2_) = *G*_1_*G*_2_ = *G*_1_ ∧ *G*_2_ (Table 7). The union operation between the partitions expresses the logical operation OR, denoted by ∨, e.g. ${f}_{14}\left({G}_1,{G}_2\right)={G}_1{G}_2\cup {G}_1\overline{G_2}\cup \overline{G_1}{G}_2={G}_1\vee {G}_2$. Figure 6 depicts *f*_*i*_(*G*_1_, *G*_2_) for *i =* 3, 6, 8, and 14, corresponding to the PC logics $\overline{G_1}$, *XOR*(*G*_1_, *G*_2_), *AND*(*G*_1_, *G*_2_), and *OR*(*G*_1_, *G*_2_), respectively. Note that there is a fundamental difference between the PC and the Boolean logics. The PC logic performs the logical operation on the continuous data, and its output is not restricted to the Boolean values of 0 and 1, but, in contrast, the output is a continuous value in the interval [0, 1].

### Probabilistic and fuzzy logics

To define the logical operators for the continuous gene expression data, previous studies usually utilize the fuzzy logic [19, 22], as given in Table 8. However, we propose an alternative logic, i.e., the PC logic, which is based on the probabilistic rules. All Boolean functions can be described by the combination of three basic logical operators: AND, OR, and NOT [40]. The definitions of these basic logical operations for the case of having two regulatory genes *G*_1_ and *G*_2_ and with the expression levels ${\mathit{\exp}}_{G_1}$ and ${\mathit{\exp}}_{G_2}$ are compared in Table 8 for the PC and the fuzzy logics.

**Table 8:** The PC logic and the fuzzy logic for the regulatory effects of genes *G*_1_ and *G*_2_ on the target, utilizing continuous gene expression data. *exp*_*A*_ and *exp*_*B*_ denote the expression levels of genes *G*_1_ and *G*_2_, respectively

Logic	Probabilistic Logic Def.	Fuzzy Logic Def.
$NOT\left({\mathrm{G}}_1\right)=\overline{{\mathrm{G}}_1}$	$1-{\mathit{\exp}}_{{\mathrm{G}}_1}$	$1-{\mathit{\exp}}_{{\mathrm{G}}_1}$
*AND*(G_1_, G_2_) = G_1_ ⋀ G_2_	${\mathit{\exp}}_{{\mathrm{G}}_1}\ast {\mathit{\exp}}_{{\mathrm{G}}_2}$	$\mathit{\min}\left({\mathit{\exp}}_{{\mathrm{G}}_1},{\mathit{\exp}}_{{\mathrm{G}}_2}\right)$
*OR*(G_1_, G_2_) = G_1_ ⋁ G_2_	${\mathit{\exp}}_{G_1}+{\mathit{\exp}}_{G_2}-{\mathit{\exp}}_{G_1}\ast {\mathit{\exp}}_{{\mathrm{G}}_2}$	$\mathit{\min}\left(1,{\mathit{\exp}}_{{\mathrm{G}}_1}+{\mathit{\exp}}_{{\mathrm{G}}_2}\right)$

In the case of *k* = 1, only gene G_1_ is in the causal set of the target gene T. Accordingly, Eq. (1) results in *f*_0_(G_1_) = 0, ${f}_1\left({\mathrm{G}}_1\right)=\overline{{\mathrm{G}}_1}=1-{\exp}_{G_1}$, ${f}_2\left({\mathrm{G}}_1\right)={\mathrm{G}}_1={\mathit{\exp}}_{G_1}$, and *f*_3_(G_1_) = 1, where *f*_1_(G_1_) and *f*_2_(G_1_) indicate the inhibitory and activatory effects of gene G_1_ on *T*, respectively (see Fig. 6a). By applying probabilistic logics, the output of 16 possible PC logic functions for *k* = 2 are represented in Table 7. The PC logic function *f*_*i*_(G_1_, G_2_, …, G_k_) is just an estimator of the probability of the activation of *T*, i.e., *exp*_*T*_.

### Likelihood function

Each PC logic function *f*_*i*_(G_1_, G_2_, …, G_k_) provides an estimate of the expression level of the target gene *T*. However, there are ${2}^{2^k}$ different PC logic functions for *k* regulatory genes influencing the target. Therefore, we need to evaluate the likelihood that these PC logic functions will predict the expression level of *T*. To achieve this goal, we suppose that the expression level of *T* follows a beta distribution with parameters *α* and *β*:

4 $$ pdf(T)=\frac{\Gamma \left(\alpha +\beta \right)}{\Gamma \left(\alpha \right)\Gamma \left(\beta \right)}{T}^{\alpha -1}{\left(1-T\right)}^{\beta -1}, $$

where, 0 ≤ *T* ≤ 1. We know that in this beta distribution, the expected value of the expression level is $E(T)=\frac{\alpha }{\alpha +\beta }$. Assuming *f*_*i*_(.) as an unbiased estimator of the target’s expression level, we obtain

5 $$ E(T)=\frac{\alpha }{\alpha +\beta }={f}_i(.) $$

In addition, considering *α* + *β* = *c*, where *c* is a constant, the model parameters are estimated as follows:

6 $$ \alpha ={cf}_i(.),\mathrm{and}\ \beta =c\left(1-{f}_i(.)\right). $$

To avoid getting zero parameters when *f*_*i*_(.) is either 0 or 1, a small value is added to the estimated *α* and *β* in Eq. (6). Then, for *n* gene expression samples, the logarithm of the likelihood function is

7 $$ \log (likelihood)=n\Gamma (c)-{\sum}_{s=1}^n\left[\log \Gamma \left(c{f}_i^s(.)\ \right)+\mathit{\log}\Gamma \left(c-{cf}_i^s(.)\right)+\left(c{f}_i^s(.)-1\right)\log \left({T}_s\right)+\left(c-c{f}_i^s(.)-1\right)\log \left(1-{T}_s\right)\right], $$

in which, *T*_*s*_ indicates the expression level of the *s-*th sample of the target gene, and ${f}_i^s(.)$ is the PC logic function computed for the corresponding sample.

The c value calibrates the variance of the target gene expression (*T*) given its regulators, in the beta distribution. As *T* values are modelled separately for each sample, i.e., *T* is expected to be close to *f*_*i*_(.), we consider a large value for c to assure a low deviation from *f*_*i*_(.).

Equation 7 is maximized w.r.t the binary variables ${\left({i}_{2^k-1},\dots, {i}_2,{i}_1,{i}_0\right)}_2$, representing the on/off state of the 2^*k*^ partitions in the venn diagram of the *k* regulatory genes. For this purpose, the current version of the LogicNet evaluates the likelihood under all possible values of these binary variables, i.e., the exact solution.

For the microarray data, the min-max feature scaling is applied to normalize the expressions into the [0, 1] interval, e.g., for a gene A:

$$ \frac{{\mathit{\exp}}_A-\mathit{\min}\left({\mathit{\exp}}_A\right)\ }{\mathit{\max}\left({\mathit{\exp}}_A\right)-\mathit{\min}\left({\mathit{\exp}}_A\right)} $$

The LogicNet is originally proposed to reconstruct the logic based GRNs, from microarrays. However, the count distribution in the RNA-seq data can also be transformed to a distribution close to the Gaussian distribution, using the voom transformation [41]. Then, the min-max feature scaling is applied [41].

### Bayesian information criterion (BIC)

The LogicNet computes the likelihood for the expression level of the target gene by considering *k* regulatory genes. However, increasing the number of regulatory genes may potentially result in model over-fitting. Here, we use the Bayesian Information Criterion (BIC) [42] to strike the right balance between improving the model fitting (likelihood) and making the model more complex. BIC is defined as follows [42]:

8 $$ BIC=-2 Loglikelihood(Model)+ number\ of\ parameters\ast Log(n) $$

In the case of having *k* regulatory genes, we consider 2^*k*^ parameters in the model that are associated with 2^*k*^ partitions of the Venn diagram, where each partition either exists or does not exist in the *f*_*i*_(G_1_, G_2_, …, G_k_). To this end, the PC logic function with a minimum BIC is considered for each target gene.

### Bayes factor (BF)

The PC logic corresponding to the minimum BIC is not necessarily biologically significant and meaningful. To distinguish between random and biologically meaningful logics, the LogicNet applies the Bayes Factor (BF) [43] to test the likelihood significance of the PC logic function with a minimum BIC. The BF is the ratio of the likelihood probabilities for two competing hypotheses as follows:

9 $$ BF=\frac{Pro\left( Target\ Gene\ expression\ Data|{M}_1\right)}{Pro\left( Target\ Gene\ expression\ \mathrm{D} ata|{M}_0\right)}, $$

where M_1_ is the PC logic function with the minimum BIC and indicates the causal relationships between the regulatory and target genes. M_0_ is a random logic without a biological significance. Based on the Bayesian literature, a value of *BF* > 100 means that compared to M_0,_ M_1_ is decisively supported by data.

**Fig. 7:**
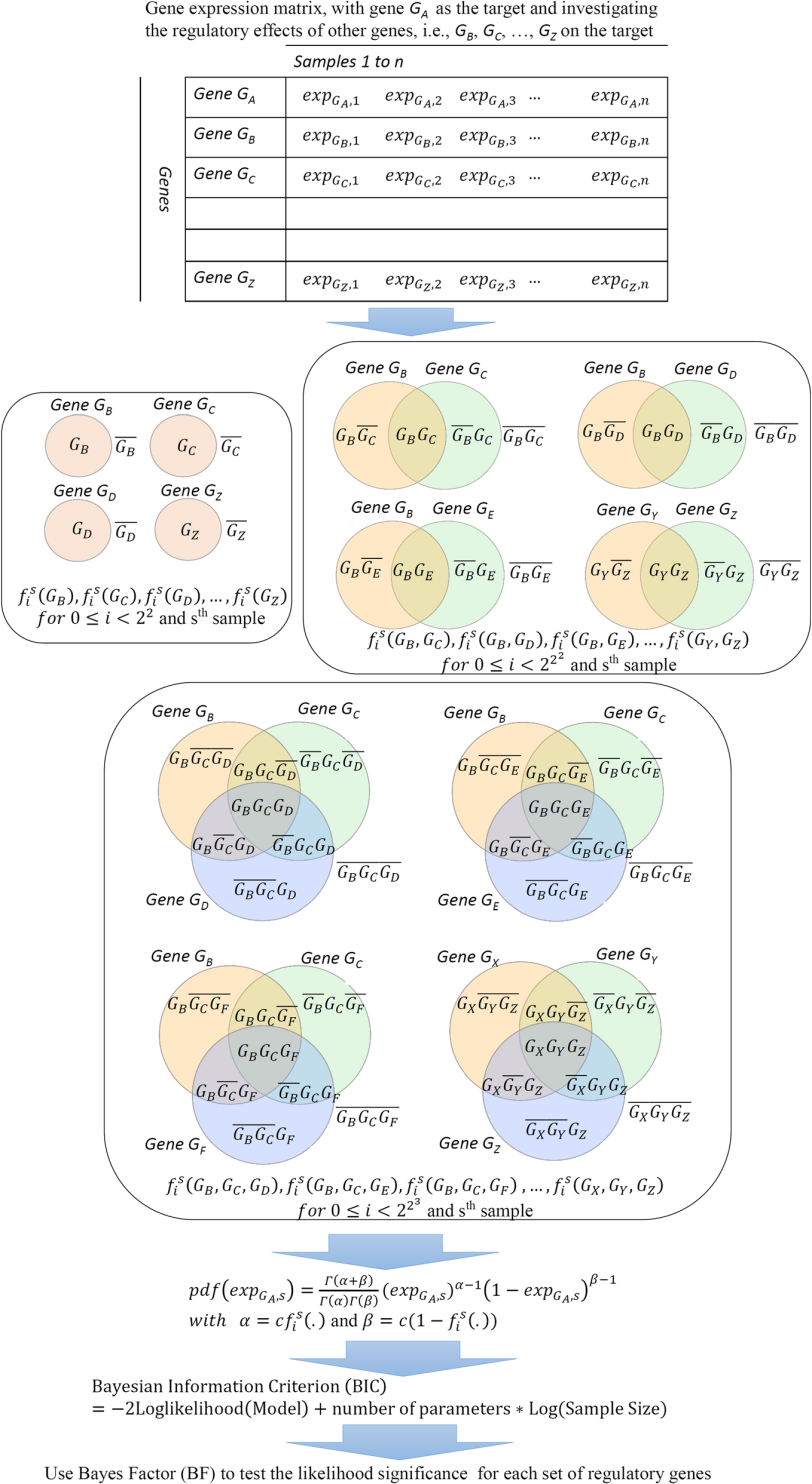
Workflow diagram of the LogicNet to reconstruct the Gene Regulatory Network. Assume n samples are drawn from the expression level of genes *G*_*A*_, *G*_*B*_, …, and *G*_*Z*_. Each time, one gene is considered as the target, and the regulatory effect of other genes on the target is investigated. Here, gene *G*_*A*_ is considered as the target. Logic functions consisting of k regulatory genes are constructed for k = 1, 2, 3, and the target gene expression likelihoods are evaluated under different logic functions. To calculate the likelihood, the target’s gene expression is modeled by using a beta distribution whose parameters are identified based on the logic function between the regulatory genes. Then, the Bayesian Information Criterion (BIC) is applied to strike the right balance between the likelihood and model complexity (the number of the regulatory genes). For each target gene, the likelihood significance in the logic function with the lowest BIC is further evaluated by using the Bayes Factor (BF). Only logic functions which are decisively supported by the target gene expression data (with BF > 100) are considered to be significant

The overall workflow of the LogicNet is depicted in Fig. 7. From genes *G*_*A*_, *G*_*B*_, …, and *G*_*Z*_, one gene at a time is considered as the target. Considering gene *G*_*A*_ as the target and *k* = 1, 2, 3, … genes as its regulators, the PC logic functions *f*_*i*_(.) are constructed for different subsets of genes *G*_*B*_, …, and *G*_*Z*_. Then, the likelihood of the expression level of the target gene (i.e., gene *G*_*A*_) is calculated under each PC logic function. BIC is applied to strike the right balance between the likelihood and model complexity, i.e., the number of the regulatory genes. The likelihood significance in the PC logic function with the lowest BIC is consequently evaluated by using the BF. This process is repeated for each gene as the target. The maximum of *k* in this study is 4.

### The LogicNet integrative performance for directed edges and logic functions

To evaluate the integrative performance of the LogicNet for the simultaneous detection of the directed edges and the logic functions, we apply a new measure in which we consider a TP if the regulatory genes and the active partition in the Venn diagram are both correctly predicted. In addition, we consider an FP if either the regulatory genes or the active partitions in the Venn diagram are predicted falsely. All other predictions are considered as FN. For example, in the case of *f*_14_(G_1_, G_2_) = G_1_ ∪ G_2_ in Fig. 6, three partitions G_1_G_2_, ${\mathrm{G}}_1\overline{{\mathrm{G}}_2}$ and $\overline{{\mathrm{G}}_1}{\mathrm{G}}_2$ of the Venn diagram are active, and therefore, we consider a TP for the correct prediction of each partition and a FP if either gene G_1_ or gene G_2_ or the corresponding active partitions are falsely predicted.

**Data and LogicNet availability Project name:** LogicNet. **Project Home Page:**https://github.com/CompBioIPM/LogicNet.

**Operating System:** Windows and Linux (× 86 and × 64 versions).

**Programming Language:** Designed in R.

**License:** Freely available under R-3.0.0 or higher versions.

**Any restrictions to use by non-academics:** none.

## Supplementary information

**Additional file 1: Supplementary Table 1.** provides more details about the logic function calls that are made by the LogicNet in the regulatory factor-target gene interactions, in the yeast database.

## Data Availability

The datasets supporting the conclusions of this article are available in the https://github.com/CompBioIPM/LogicNet repository.

## Abbreviations

ACC: Accuracy
BF: Bayes Factor
BIC: Bayesian Information Criterion
FN: False Negative
FP: False Positive
FPR: False Positive Rate
GRNs: Gene Regulatory Networks
MCC: Matthews’s Coefficient Constant
MI: Mutual Information
PCA: Principle Component Analysis
PC-Logic: Probabilistic Continuous Logic
PPV: Positive Predictive Value
RFs: Regulatory Factors
ROC: Receiver Operating Characteristic
T: Target gene
TN: True Negative
TP: True Positive
TPR: True Positive Rate
